# Exploring Research Priorities of Parents Who Have Children With Down Syndrome, Cleft Lip With or Without Cleft Palate, Congenital Heart Defects, or Spina Bifida Using ConnectEpeople: A Social Media Coproduction Research Study

**DOI:** 10.2196/15847

**Published:** 2019-11-25

**Authors:** Marlene Sinclair, Julie EM McCullough, David Elliott, Anna Latos-Bielenska, Paula Braz, Clara Cavero-Carbonell, Anna Jamry-Dziurla, Ana João Santos, Lucía Páramo-Rodríguez

**Affiliations:** 1 Institute of Nursing and Health Research Ulster University Newtownabbey, Northern Ireland United Kingdom; 2 Redburn Solution Ltd Belfast United Kingdom; 3 Department of Medical Genetics, Poznan University of Medical Sciences Poznan Poland; 4 Epidemiology Department, National Institute of Health Doctor Ricardo Jorge Lisbon Portugal; 5 Rare Diseases Research Unit, Foundation for the Promotion of Health and Biomedical Research in the Valencian Region Valencia Spain; 6 Public Health Research Centre, National School of Public Health, Nova University Lisbon Lisbon Portugal

**Keywords:** e-forum, social media, Web-based survey, Facebook, STAI, Down syndrome, cleft lip with or without cleft palate, congenital heart defects, spina bifida, parents, ocularcentrism, coproduction

## Abstract

**Background:**

Using social media for research purposes is novel and challenging in terms of recruitment, participant knowledge about the research process, and ethical issues. This paper provides insight into the recruitment of European parents of children with specific congenital anomalies to engage in coproduction research by using social media. Secret Facebook groups, providing optimal security, were set up for newly recruited research-aware parents (RAPs) to communicate privately and confidentially with each other and for the research team to generate questions and to interpret findings.

**Objective:**

This study aimed to use social media for the recruitment and engagement of parents in research and to determine the research priorities of parents who have children with Down syndrome, cleft lip with or without cleft palate, congenital heart defects, and spina bifida.

**Methods:**

The design was exploratory and descriptive with 3 phases. Phase 1 included the recruitment of RAPs and generation of research questions important to them; phase 2 was a Web-based survey, designed using Qualtrics software, and phase 3 included analysis and ranking of the top 10 research questions using an adapted James Lind Alliance approach. Simple descriptive statistics were used for analysis, and ethical approval was obtained from the Ethics Filter Committee of the Institute of Nursing and Health Research, Ulster University.

**Results:**

The recruitment of 32 RAPs was a sensitive process, varying in the time taken to consent (mean 51 days). However, parents valued the screening approach using the State-Trait Anxiety Inventory as a measure to ensure their well-being (mean 32.5). In phase 1, RAPs generated 98 research questions. In phase 2, 251 respondents accessed the Web-based survey, 248 consented, and 80 completed the survey, giving a completeness rate of 32.3% (80/248). Most parents used social media (74/80, 92%). Social media, online forums, and meeting in person were ranked the most preferable methods for communication with support groups networks and charities. Most respondents stated that they had a good understanding of research reports (71/80, 89%) and statistics (68/80, 85%) and could differentiate among the different types of research methodologies (62/80, 78%). Phase 3 demonstrated consensus among RAPs and survey respondents, with a need to know the facts about their child’s condition, future health, and psychosocial and educational outcomes for children with similar issues.

**Conclusions:**

Social media is a valuable facilitator in the coproduction of research between parents and researchers. From a theoretical perspective, ocularcentrism can be an applicable frame of reference for understanding how people favor visual contact.

## Introduction

### Background

The European Commission [1] highlights the value of using social media for communication and engagement with the public and acknowledges that it is a “beneficial tool to connect with others” and to “find new research partners.” Parents require health-related information about their children [2], and an increasing number seek this information from the internet and on social media platforms [3-5], with many going to online forums to discuss specific issues [6]. This is particularly true for parents who have a child with a chronic health condition [7]. Parents strongly feel that they can and must have a voice in health and education research that will have a positive impact on their child’s everyday life [8,9]. Across Europe, awareness of the benefits of patient and public involvement (PPI) in health care research is rapidly increasing [10]. Lander et al [11] has identified a number of benefits of actively engaging with service users, including the development of research goals that are congruent with those of the public and “assessing the impact and value of health technologies and health services.” However, there remains wide variation among countries in the opportunities to do so [10]. In the United Kingdom, PPI in research refers to researchers and patients, carers, and the public working in collaborative partnership to add value to the research process in an accessible and meaningful way [12]. The UK National Institute for Health Research (NIHR) incorporates and funds INVOLVE [12] to “support active public involvement in the National Health Service, public health and social care research.” “INVOLVE defines public involvement in research as research being carried out ‘with’ or ‘by’ members of the public rather than ‘to,’ ‘about’ or ‘for’ them” [12].

Social media platforms can provide the basis for reciprocal, real-time discussion and sharing of valuable, high-quality information among members with a strong *human-to-human connection* [13]. Russell et al [14] developed a study to connect parents participating in research and researchers in Canada. Using a secret Facebook group, an online discussion network was developed. The group was built by and for parents of children with special needs working with researchers to develop relevant research questions and priorities. The families involved stated that it was a valuable resource for social support and sourcing information in a secure and private way. They felt that the platform provided them with an opportunity to have their voice heard and this was empowering. Researchers involved in the study reported that they were able to connect and discuss with parents directly, which gave them a clearer understanding of the daily life and struggles of families who have a child with special needs [14].

From a theoretical perspective, understanding how and why people value technology for its combination of *electronic touching* and instantaneous access to *visible data* is an important determinant in their preference for the use of social media [15]. The term *ocularcentrism* has become familiar to researchers in social media and is a phenomenon that is built on the theory that *seeing* is believing [16]. Messages, communicated using technology that is embedded in social media with illustrative and graphical sophistication, optimize the visual representation of data in three dimensions, word, text, and video, making it more powerful, believable, and acceptable.

### Objectives

**Figure 1:**
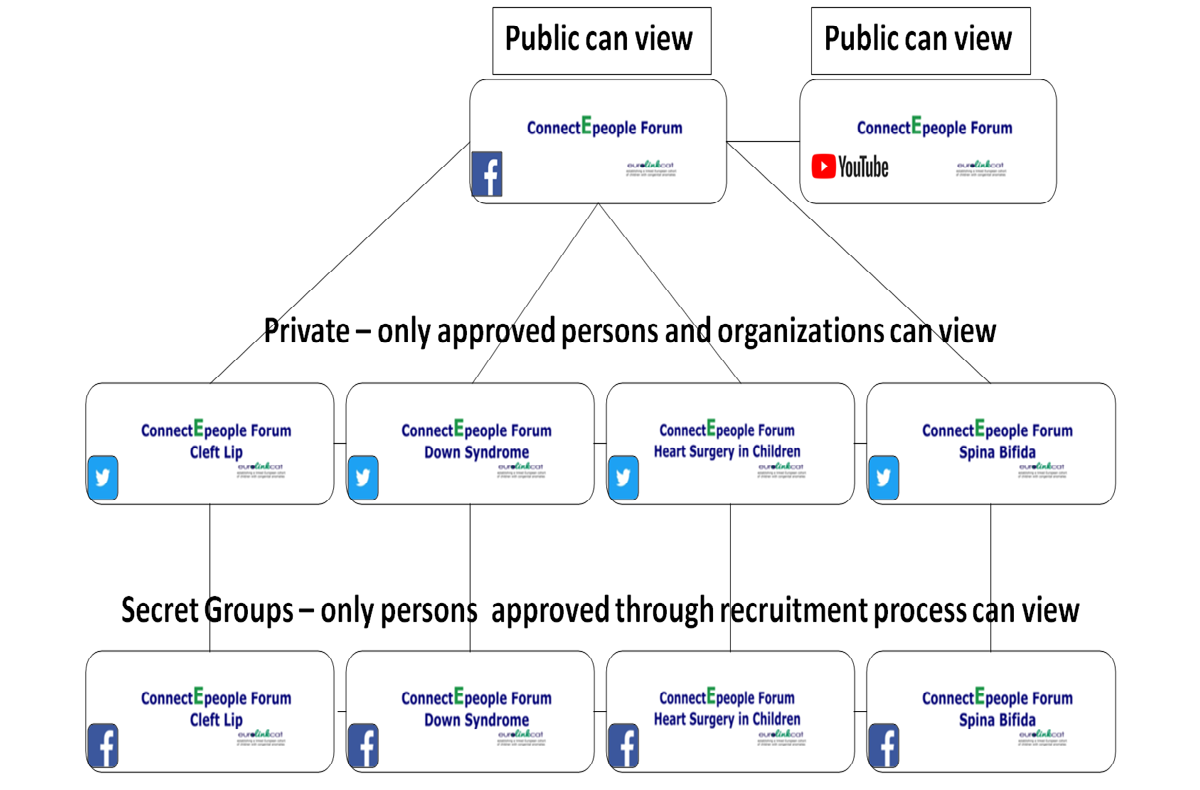
The ConnectEpeople electronic forum structure.

Establishing a linked European Cohort of Children with Congenital Anomalies (EUROlinkCAT) is a European project with a number of aims, one of which is connecting researchers and families of children with specific congenital anomalies (CAs), such as Down syndrome (DS), cleft lip with or without cleft palate (CLP), congenital heart defects (CHD), and spina bifida (SB), under the banner of ConnectEpeople. The aim of this project was to actively involve parents in setting research priorities and ensuring that research results are disseminated in a meaningful way by establishing a sustainable electronic forum (e-forum), called ConnectEpeople, to provide regional, national, and international support to families through maintaining the links between the European Surveillance of Congenital Anomalies’ (EUROCAT) registries and families [17]. Therefore, this online forum was designed to maximize public and professional engagement in research by establishing a public Facebook page, 4 private Twitter accounts, a YouTube channel, and 4 secret Facebook groups as a cohesive moderated platform: the ConnectEpeople e-forum (Figure 1).

The aim of ConnectEpeople was to use social media for recruitment and engagement of parents in research and to determine the research priorities of parents who have children with DS, CHD, CLP, and SB.

## Methods

### Overview

The design was exploratory and descriptive and used a mixed methods approach in 3 phases. Parents who had a child with 1 of the 4 CAs were actively recruited via social media to participate in an online research forum using secret Facebook groups. Participating parents were known as research-aware parents (RAPs). Working in partnership with RAPs, an online introduction to the overall project objectives of EUROlinkCAT [18] was made available, and participants were asked to familiarize themselves with the content at the beginning of the study. RAPs provided the research team with questions that were important to them in relation to their child’s condition. A Web-based survey was developed, which was open to any parent of a child with 1 of the 4 conditions. The findings from the survey were analyzed and discussed with the RAPs for further feedback and clarification.

### Study Phases

#### Phase 1: Parent Engagement and the Generation of Research Questions

##### Parent Engagement and Recruitment

Parent support organizations across 9 European countries identified by the research team were contacted [19]. Those who agreed to act as gatekeepers provided information about the ConnectEpeople project to their members via their social media profiles, via group newsletters, in person, on the telephone, and via other communication networks. The researcher contacted the parents, provided them with the participant information sheet, and arranged a time to discuss details about the project and the parents’ research needs face-to-face using Skype, WhatsApp, or FaceTime, via video chat on Facebook Messenger, or meeting in person.

Parents living in Croatia, France, Germany, Italy, Netherlands, Poland, Portugal, Spain, and the United Kingdom interested in joining the ConnectEpeople project were invited to do so if they met the following inclusion criteria: they were a parent of a child with CLP, DS, CHD, or SB; a member of a recognized CA parent organization; their child was aged between 1 and 11 years; they had access to social media; and they were able to understand English. Potential participants were screened to ensure they were in good psychological health to participate in the study. The screening procedure involved a self-completion measure of their current anxiety level by completing the State-Trait Anxiety Inventory (STAI) [20] online. Various reliability and validity tests have been conducted on the STAI and have provided sufficient evidence that it is an appropriate, reliable, and adequate measure for studying anxiety in research and clinical settings [21] and indicates anxiety levels for a single point in time. The average STAI scores for working adult (aged 19-69 years) females is 35.2 (SD 10.61) and males is 35.72 (SD 10.40); the score is also dependent on the age of the respondent [20]. In a South American validation study of the STAI and the Beck Depression Inventory, the mean STAI scores for anxious participants was 52.8 (SD 11.4) [22]. Therefore, given the nature of the medical conditions under consideration, STAI anxiety scores above the average for this population were expected, and the cutoff score was set at 65. Once parents completed the STAI, which was available in 13 languages, and received their score, they were asked to sign a consent form and a project-specific social media policy that outlined the standards ConnectEpeople required participants to observe when using social media. Following this, parents were invited to join the ConnectEpeople secret Facebook group. Secret Facebook groups are not visible to the public, and membership was by invitation only from the moderators (MS/JMcC). In the group, communication was restricted to the specific CA cohort, thus increasing confidentiality and encouraging open dialogue. Recruitment ran from January 2018 to March 2019.

##### Generation of Research Questions

Research questions for inclusion in the Web-based survey were identified with RAPs using Facebook, WhatsApp, Skype, and video chat, during the ConnectEpeople recruitment process. RAPs used email, telephone, and discussion in secret Facebook groups to identify questions. During the discussions with RAPs, the researcher provided details about the EUROlinkCAT project’s aims and specific research objectives to collect data on *education*, *morbidity*, and *survival* and the role of ConnectEpeople. All RAPs were asked the open question “Is there a research question that you already have that you would like an answer to?” Questions generated through this process were compiled and reviewed by 2 researchers for inclusion or exclusion in the project survey.

#### Phase 2: Survey Development

A Web-based survey was developed using Qualtrics software. The survey was designed in English and translated into Polish, Portuguese, and Spanish. The translation process included changes necessary to ensure that the survey was culturally, socially, and regionally acceptable to the respondents. The survey was reviewed for face and content validity with 12 researchers with experience in using Web-based surveys and/or CAs and 7 RAPs, all of whom were based in Poland, Portugal, or the United Kingdom. Following this pilot stage, minor amendments were made. The duration and the complexity of the survey were reduced by applying survey logic, and thus, only the questions that were relevant to each respondent, based on previous answers, were displayed. The incorporation of a back button allowed respondents to change their answers.

The Web-based survey comprised 62 items, and duplicate entries were avoided by preventing users’ access to the survey twice. In addition to seeking verification on research questions generated by RAPs in phase 1, on a 4-point Likert scale of *really important*, *important*, *not sure*, or *definitely not important*, additional questions were focused on obtaining information on demographics, modes of communication with support groups, research knowledge, and use of the internet for research.

Respondents were asked to rate questions on the use of the internet for research-related searches on a 5-point Likert scale of *strongly agree*, *somewhat agree*, *neither agree nor disagree*, *somewhat disagree*, and *strongly disagree*. The survey finished with an open request for parents to identify a question on their research needs related to their child’s conditions.

All survey data and personal data were stored in 1 password-protected Qualtrics account and an appropriate password-protected computer on a password-protected network within the Institute of Nursing and Health Research, Ulster University, United Kingdom.

#### Phase 3: Data Analysis of Survey

Following the closure of the survey, all data were cleaned, checked for errors, and analyzed within the Qualtrics system using simple descriptive statistics. For analysis purposes, the Likert items were recoded into 3 categories; research questions generated by RAPs that were reported as *really important* and *important* were combined. Similarly, for the questions related to research needs, responses of *strongly agree* and *somewhat agree* were combined and *strongly disagree* and *somewhat disagree* were combined. Following the identification of the most important research questions, the RAPs in the 4 ConnectEpeople secret Facebook groups were consulted to seek consensus on relevancy and ranking.

The James Lind Alliance (JLA) [23] is a research initiative that brings patients, carers, and clinicians together in “Priority Setting Partnerships (PSPs) to identify and prioritize the Top 10 unanswered questions or evidence uncertainties that they agree are the most important” [23]. The overall aim was to ensure that researchers and funding bodies understand the key health questions that the patients and the public view as research priorities. Therefore, drawing from the JLA guidelines, the research team sought to use an adapted approach to develop a list of parent’s top 10 research priorities. Survey respondents’ rating of the proposed research questions were reviewed and confirmed for relevancy by the RAPs in their secret Facebook groups.

### Ethical Considerations

Ethical approval for the study was obtained from the Ethics Filter Committee of the Institute of Nursing and Health Research, Ulster University, on November 21, 2017.

## Results

### Research-Aware Parents

**Figure 2:**
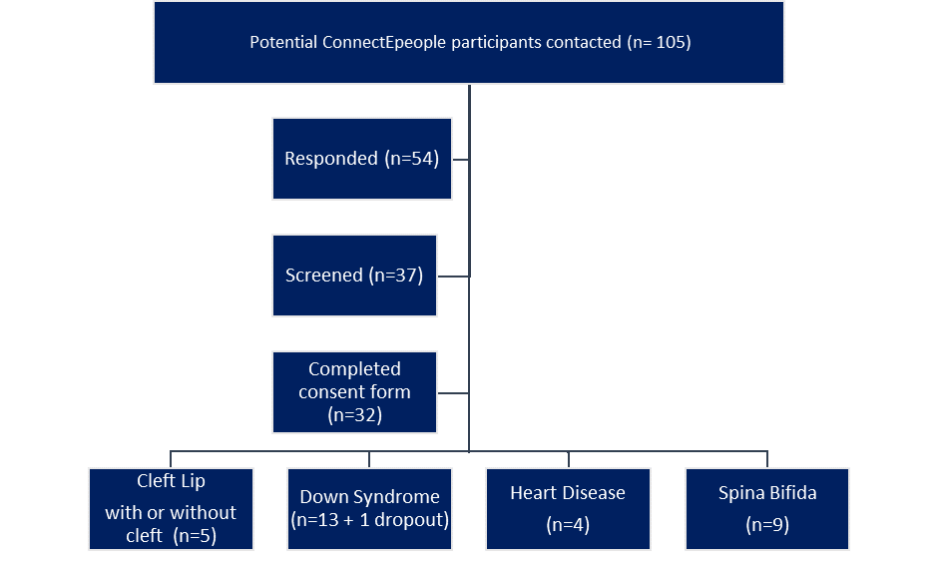
Recruitment of research-aware parents to the ConnectEpeople project.

Recruitment took place online for all participants and was welcomed by parents who found working online was easier for them. Following the notification of interest in ConnectEpeople, 105 parents were contacted, of whom 54/105 (51.4%) responded, 38/105 (36.2%) completed the screening process, and 32/105 (30.5%) entered the secret Facebook element of the ConnectEpeople e-forum (Figure 2). RAPs came from 7 countries: Croatia, Germany, Italy, Poland, Portugal, Spain, and the United Kingdom. The average duration for recruitment from first contact by the parent until entry into the secret Facebook groups was 51 days.

The inclusion of the STAI for screening purposes demonstrated to parents that the ConnectEpeople research team cared about their health and well-being:

…no one ever cared about my mental health before.

RAP, DS, Poland

The mean STAI scores across all 4 CAs were below the cutoff score identified for use in this project, and all parents who were screened were invited to take part. The average STAI score across all 4 groups was 32.5; the lowest scores were for RAPs whose children had CLP, and the highest scores were for those with a child with CHD (Multimedia Appendix 1). No parent was excluded based on their STAI score.

A total of 98 questions were identified through conversations with RAPs for inclusion in the survey; 28 questions were generated by RAPs who had a child with DS, 23 questions by RAPs who had a child with CLP, 22 questions by RAPs who had a child with SB, and 25 questions by RAPs who had a child with CHD. In the secret Facebook groups, RAPs discussed the survey respondents’ rating of the questions. Rating of research questions demonstrated a high level of consistency between RAPs and survey respondents for all 4 CA groups.

### Survey Respondents

A total of 251 parents accessed the survey, 248 consented to take part, and 227 completed the first page of the survey. Respondents were from the following 17 countries: Bulgaria, Croatia, Germany, India, Ireland, Lithuania, Netherlands, Panama, Peru, Poland, Portugal, South Africa, Spain, Turkey, the United Arab Emirates, the United Kingdom, and the United States. Overall, 100 partially completed surveys from parents of a child with DS (35/100), SB (39/100), CL*P* (16/100), and CHD (10/100) provided sufficient data for analysis. Moreover, 80 respondents fully completed the survey, giving a completeness rate of 32.3% (80/248). Table 1 illustrates the demographic profile and use of social media.

**Table 1:** Demographic details of ConnectEpeople survey respondents who fully completed the survey and their use of social media (N=80).

Characteristic		Value	
**Parent, n (%)**			
	Mother		75 (94)
	Father		5 (6)
Age (years), mean (SD)		38 (6.77)	
**Relationship status, n (%)**			
	With partner		74 (93)
	No partner		6 (7)
**Member of a parent support organization, n (%)**			
	Yes		66 (83)
	No		14 (17)
**Use of social media, n (%)**			
	Facebook		74 (93)
	WhatsApp		66 (83)
	Twitter		20 (25)
	Snapchat		16 (20)
	Instagram		6 (8)
**Able to speak English, n (%)**			
	Yes		79 (99)
	No		1 (1)
**Educational attainment, n (%)**			
	Secondary education		4 (5)
	Diploma		14 (18)
	Undergraduate		29 (36)
	Postgraduate		33 (41)
**Condition of child, n (%)**			
	Down syndrome		28 (35)
	Spina bifida		28 (35)
	Cleft lip with or without cleft palate		16 (20)
	Congenital heart defects		8 (10)
**Country of residence, n**			
	United Kingdom		32 (40)
	Portugal		14 (18)
	Poland		12 (15)
	Ireland		9 (11)
	Germany		2 (2.5)
	Netherlands		2 (2.5)
	Spain		2 (2.5)
	Bulgaria		1 (1)
	Croatia		1 (1)
	India		1 (1)
	Lithuania		1 (1)
	Panama		1 (1)
	Turkey		1 (1)
	United States		1 (1)

### Use of Social Media and the Internet

In total, 73/80 (91%) respondents used a number of methods to communicate with support groups; Multimedia Appendix 2 demonstrates their communication preferences. Social media was preferred because of its accessibility, speed of contact, visual choices, ease of use, multiple links, and 24-hour availability. Mobile phones were the main method of connecting to the internet (62/80, 78%).

Face-to-face discussion was convenient and preferred for personal and confidential conversations, and discussion forums were the most popular because of the opportunity for personal sharing among people who have similar issues of concern. This was supported by comments from respondents and RAPs:

Using technology and social media is the way forward and the best way to get parents involved as practically it can be difficult to have the time to meet face to face or at particular times, this is more flexible.

RAP, SB, United Kingdom

It gives the broader range of possibilities, you can not only read and watch but you can contribute, you can share links.

Respondent, DS, Poland

Most respondents felt that it was important for parents to be able to understand basic research (71/80, 89%). The majority had a good understanding of research reports (71/80, 89%) and statistics (68/80, 85%) and could differentiate between different types of research methodologies (62/80, 78%).

### Research Questions

Tables 2-5 show the top 10 questions for each CA ranked by importance based on the survey results. Further thematic analysis resulted in the identification of the following 4 main subthemes:

- Facts: All parents were concerned about the facts concerning their child’s condition; however, the ranking order demonstrated that parents with a child who had CHD considered these questions to be top priority.
- Health: Most parents were concerned about a wide range of health issues, and this was ranked the highest by parents who had children with DS.
- Education: There was consensus among all parents about the importance of education, and this was particularly important in the ranking for parents of children with SB.
- Psychosocial impact: This issue was ranked to be of equal importance across all groups.

**Table 2:** Ten most important research questions of ConnectEpeople survey respondents with children who have Down syndrome (N=35).

Question	Really important/important, n (%)	Not sure, n (%)	Definitely not important, n (%)
How can I maximize my child’s educational attainment?	34 (97)	1 (3)	0 (0)
What dietary supplements should my child be taking?	34 (97)	1 (3)	0 (0)
Does exercise enhance the immune system of children with Down syndrome?	33 (94)	1 (3)	1 (3)
Would early intervention, eg, tummy time, creeping, and crawling, enhance my child’s development?	33 (94)	1 (3)	1 (3)
How many children, with the same condition as my child, go to mainstream school?	32 (91)	1 (3)	2 (6)
Where would I find specialized information such as video clips of parents feeding a baby with my child’s condition?	32 (91)	0 (0)	3 (9)
Is obesity a problem with my child’s condition?	32 (91)	0 (0)	3 (9)
What is the latest genetic research relating to my child’s condition?	32 (91)	3 (9)	0 (0)
What is the psychosocial impact of my child’s condition on my child and our family?	32 (91)	1 (3)	2 (6)
What complementary therapies are beneficial for my child?	32 (91)	2 (6)	1 (3)

**Table 3:** Ten most important research questions of ConnectEpeople survey respondents with children who have spina bifida (N=39).

Question	Really important/important, n (%)	Not sure, n (%)	Definitely not important, n (%)
How many children have surgery and how many survive?	37 (95)	2 (5)	0 (0)
What is the psychosocial impact of my child’s condition on my child and our family?	37 (95)	2 (5)	0 (0)
What is the normal milestone development for a child with the same condition as my child?	36 (92)	2 (5)	1 (3)
If my child has to take time out of school, will their education continue?	36 (92)	3 (8)	0 (0)
How can I maximize my child’s educational attainment?	36 (92)	3 (8)	0 (0)
What complementary therapies are beneficial for my child?	36 (92)	2 (5)	1 (3)
What dietary supplements should my child be taking?	35 (90)	3 (8)	1 (2)
What devices or products are the best to buy for my child at different life stages?	35 (90)	3 (8)	1 (2)
How many children, with the same condition as my child, go to mainstream school?	35 (90)	3 (8)	1 (2)
What type of operations are available for babies in the womb to reduce the effect of their condition?	34 (87)	4 (10)	1 (3)

**Table 4:** Ten most important research questions of ConnectEpeople survey respondents with children who have cleft lip with or without cleft palate (N=16).

Question	Really important/important, n (%)	Not sure, n (%)	Definitely not important, n (%)
What is the rate of reoccurrence of cleft lip with or without cleft palate among siblings?	16 (100)	0 (0)	0 (0)
Are there lactation consultants with expertise in supporting parents who have a child like mine?	16 (100)	0 (0)	0 (0)
What are the genetic and environmental causes of cleft lip with or without cleft palate?	16 (100)	0 (0)	0 (0)
Where would I find specialized information such as video clips of parents feeding a baby with my child’s condition?	16 (100)	0 (0)	0 (0)
What is the best age for children with a cleft to have surgery?	16 (100)	0 (0)	0 (0)
What is the latest genetic research relating to my child’s condition?	15 (94)	1 (6)	0 (0)
How can I maximize my child’s educational attainment?	14 (88)	2 (12)	0 (0)
What complementary therapies are beneficial for my child?	14 (88)	2 (12)	0 (0)
What is the psychosocial impact of my child’s condition on my child and our family?	14 (88)	0 (0)	2 (12)
What is the normal milestone development for a child with the same condition as my child?	13 (81)	2 (13)	1 (6)

**Table 5:** Ten most important research questions of ConnectEpeople survey respondents with children who have congenital heart defects (N=10).

Question	Really important/important, n (%)	Not sure, n (%)	Definitely not important, n (%)
If my child is diagnosed with a heart condition in the womb, are there any medications I can take to help my baby?	10 (100)	0 (0)	0 (0)
Is it okay for my child to get vaccinated?	10 (100)	0 (0)	0 (0)
Is there an increased number of hospital admissions during winter with children with heart defects?	10 (100)	0 (0)	0 (0)
Is obesity a problem with my child’s condition?	10 (100)	0 (0)	0 (0)
What is the latest genetic research relating to my child’s condition?	10 (100)	0 (0)	0 (0)
How can I maximize my child’s educational attainment?	10 (100)	0 (0)	0 (0)
What is the psychosocial impact of my child’s condition on my child and our family?	10 (100)	0 (0)	0 (0)
Can you pick up heart defects during pregnancy and reduce the damage?	9 (90)	1 (10)	0 (0)
How many children have heart surgery and how many survive?	9 (90)	1 (10)	0 (0)
What age is my child likely to live to?	9 (90)	1 (10)	0 (0)

## Discussion

### Principal Findings

The key finding from this social media research is that parents with children who have CAs value social media for connecting with others and to obtain information about their child’s condition, future health, well-being, educational outcomes, and psychosocial issues. This study affirms growing parental preference for information and support via interactive social media and less interest in commonly perceived useful sources of support for parents, such as advice chat lines (online or telephone).

#### Social Media

By using secret Facebook groups, the ConnectEpeople e-forum enabled a process of engaging in a consultative dialogue online between researchers and parents living in European countries who have children with DS, CLP, SB, and CHD. Parents were able to contribute and collaborate to identify and prioritize research questions in a private, confidential, and secure online community. The Web-based survey respondents clearly identified social media as a popular and preferable method of communicating with others. Online discussion forums were important for parents to communicate and connect with others with shared life experiences (living with children who have CAs). The traditional method of connecting, namely, meeting in person, remains desirable when communicating with others; therefore, our approach of connecting online, using visual technology (WhatsApp, Skype, and other forms of video chat) to overcome distance, enabled the parents to *see* each other and connect with a person as opposed to making a connection with a text or a voice. Theoretically, this is what we value in ocularcentrism, where people need to see each other first and then they can communicate more effectively using text, etc.

Heath et al [24] identified that the choice of communication method can greatly enhance the research participant’s willingness and ability to speak openly and honestly. Flexibility in the data collection approach from RAPs adopted by the ConnectEpeople study has yielded valuable and comprehensive information on parent’s research priorities.

The ConnectEpeople survey was presented as a chance for respondents to voice their research wants and needs. To demonstrate that the parent’s views were highly valued, respondents were given the opportunity to submit a research question of their own. Analysis of the additional research questions collected in the open question revealed themes consistent with the questions presented to and prioritized by survey respondents. This suggests that still much needs to be done globally to ensure that all parents of children with CAs have timely access to robust evidence-based information that they feel they need to meet the needs of their children and provide them with the best possible opportunities, care, and treatments.

#### Ocularcentrism

From a theoretical perspective, understanding how and why people value visible data is an important determinant in their preference for the use of social media. The term *ocularcentrism* has become familiar to researchers in social media and is a behavior that is built on the theory that *seeing* is believing and that we naturally favor visual contact [16]. In this research, we see parents who use “technology that is manifested in the use of social media” possibly because of its illustrative and graphical potential to optimize visual representation in person first, then in word, text, and audio [16]. The online behavior is almost second nature to the expert user who controls the software, as if they drive a car, and after a time, the behavior techniques become so normalized and intrinsic that they are almost subconscious. Current research highlights the preferences by the public for using social media [25]. Although the findings of the ConnectEpeople survey confirm this, it sheds light on the high value that parents continue to place on traditional meeting in-person communication. Therefore, given that online video chat is widely accessible, convenient, and inexpensive, it is a valuable component to facilitate meeting in person for collaborative research with geographically distant teams. Face-to-face contact is significant in building trust and rapport and enabling team members to speak their mind. Therefore, developing opportunities for research participants in other countries to meet researchers face-to-face online will facilitate a higher level of collaboration and engagement and is in keeping with our understanding of the value we place on *seeing* not only words, text, and video but also the human person and is in keeping with our reference to ocularcentrism [16].

#### Research Priorities

We expected, based on our review of the literature, that by working with parents, very different views and needs would be expressed. Therefore, the decision was taken to use the JLA approach, which has demonstrated expertise in the area of identifying research priorities. The aim of this work was to give parents who have children with CAs a platform to voice their opinion on what issues are important to them and rank them in the order of importance.

Parents had a varied response to the identification of key research questions, and RAPs who reviewed the lists of questions were *not surprised* by the findings and agreed with the value and importance of the research questions generated and the rankings. Maintaining good health, maximizing educational attainment, and improving psychosocial aspects were among the top concerns across all 4 CAs. The areas of interest for respondents and the parents who developed the list of questions were concerned with a range of issues, eg, achieving childhood developmental milestones, infant feeding, complementary therapies, and exercise. There may be a lack of available information for parents in these areas that would resonate with the research by McHugh et al [26] who made a strong argument for collaborative research for children with chronic health conditions. Their work demonstrated that parents’ and investigators’ research needs are often incongruent and researchers do not clearly understand the issues that are important to parents. Parents and carers of children participating in research need to be involved in the process of prioritizing research questions. Morris et al [27] demonstrated the benefits of a British Academy of Childhood Disability, JLA Research PSP for children with disabilities, working with a wide range of stakeholders, including parents. Together, the multiprofessional team developed a *Top Ten List* of research priorities that led to the identification of funding opportunities from the NIHR and National Institute for Health and Care Excellence (NICE) guidelines. Therefore, the onus is placed on researchers and health professionals to address these information needs and keep abreast of parents’ changing needs.

#### Additional Findings

Although the recruitment process was lengthy, it was ethically appropriate and was a clear demonstration of our sensitivity and human caring for parents. This was confirmed when organizations and parents commented on the value of the face-to-face recruitment approach and using the STAI as a measure of well-being. We report this finding as new knowledge about the value of a screening tool such as the STAI to facilitate ethical recruitment in sensitive research cases.

### Limitations

The research limitations include the need for RAPs to be able to understand English, be able to access the survey online, have a child aged between 1 and 11 years, and be users of social media/technology for communication. In addition, 77% (62/80) of the survey respondents who fully completed the survey were educated to degree or postgraduate level. The survey was live from May 24, 2018, to October 8, 2018, over the holiday period, and the sample size was small.

### Conclusions

The use of social media and online parental engagement in research within the ConnectEpeople project enabled the identification of parent’s research priorities. Working online and using face-to-face apps and technology can build trust and foster the collaboration by exploiting multiple communication channels to maximize engagement and partnership between researchers and parents to produce accessible, meaningful, and usable information. The survey revealed that meeting in person continues to be highly valued alongside social media and discussion forums. The agreement in research priorities between the survey respondents and the RAPs and the wide geographical engagement suggest a high degree of commonality of the research wants and needs of parents of children with these CAs, regardless of global location.

## Appendix

Multimedia Appendix 1 Mean state anxiety scores (95% CI), based on the State-Trait Anxiety Inventory, of screened parents according to the congenital anomaly of their child.

Multimedia Appendix 2 ConnectEpeople survey respondents’ communication preferences with support group(s), networks, or charities (n=65).

## Abbreviations

CA: congenital anomaly
CHD: congenital heart defects
CLP: cleft lip with or without cleft palate
DS: Down syndrome
e-forum: electronic forum
EUROlinkCAT: Establishing a linked European Cohort of Children with Congenital Anomalies
JLA: James Lind Alliance
NIHR: National Institute for Health Research
PPI: patient and public involvement
PSP: Priority Setting Partnership
RAP: research-aware parent
SB: spina bifida
STAI: State-Trait Anxiety Inventory
