# Supplementary figures and images for: Exploring Research Priorities of Parents Who Have Children With Down Syndrome, Cleft Lip With or Without Cleft Palate, Congenital Heart Defects, or Spina Bifida Using ConnectEpeople: A Social Media Coproduction Research Study
Source: J Med Internet Res. 2019 Nov 25;21(11):e15847. doi: 10.2196/15847 (PMC6902131; doi:10.2196/15847)

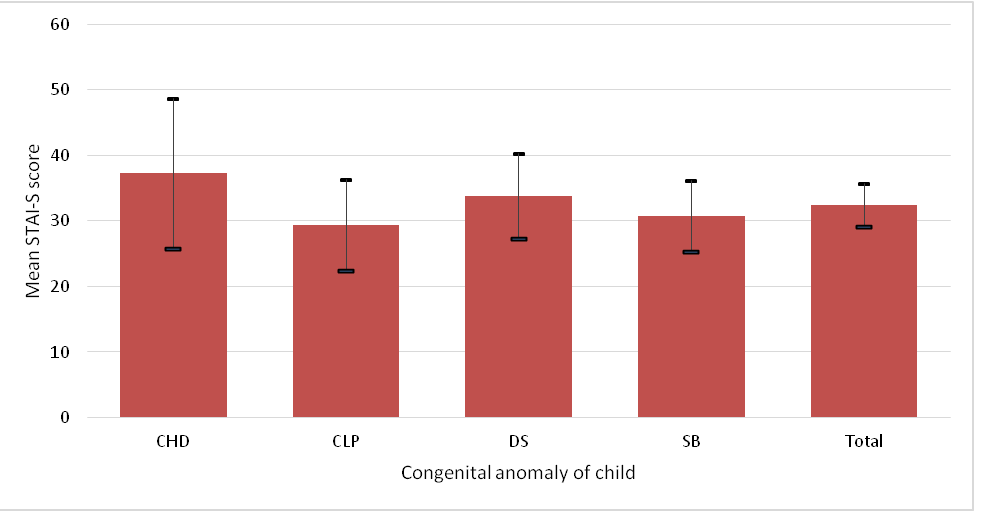

Supplement: Multimedia Appendix 1 [file jmir_v21i11e15847_app1.png]

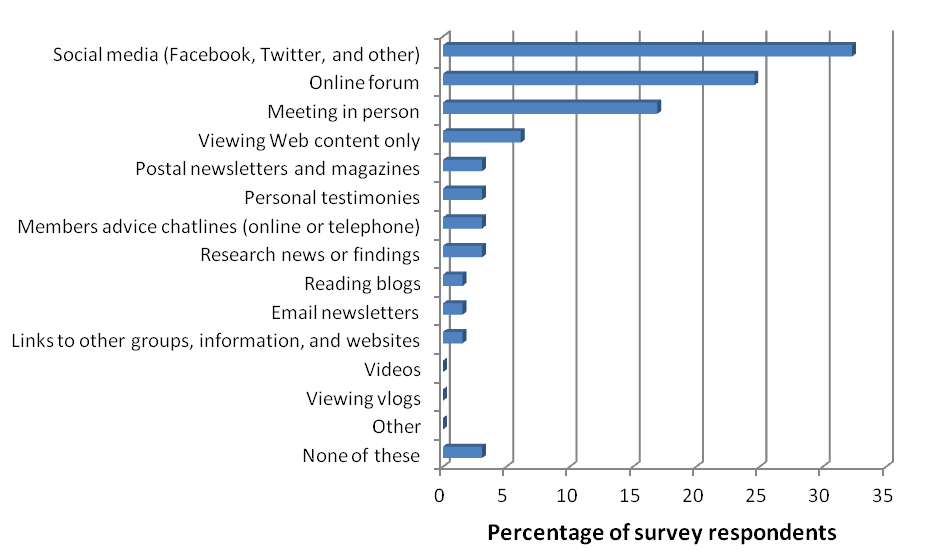

Supplement: Multimedia Appendix 2 [file jmir_v21i11e15847_app2.png]
